# Paradox of the Relationship between Cardio-Ankle Vascular Index and Ankle-Brachial Index in Patients with Lower Extremity Artery Disease

**DOI:** 10.3400/avd.avd.oa.23-00055

**Published:** 2023-11-01

**Authors:** Yoko Sotoda, Shigeki Hirooka, Hiroyuki Orita, Ichiro Wakabayashi

**Affiliations:** 1 Department of Cardiovascular Surgery, Yamagata Saisei Hospital, Yamagata, Yamagata, Japan; 2 Department of Environmental and Preventive Medicine, School of Medicine, Hyogo Medical University, Nishinomiya, Hyogo, Japan

**Keywords:** ankle-brachial index, arterial stiffness, cardio-ankle vascular index, lower extremity artery disease, toe-brachial index

## Abstract

**Objectives:** Measurements of ankle-brachial index (ABI) and toe-brachial index (TBI) are standard examinations for evaluating arterial blood flow in lower extremities and diagnosing lower extremity artery disease (LEAD). It remains to be clarified whether cardio-ankle vascular index (CAVI), a blood pressure-independent parameter of arterial stiffness, is associated with ABI and TBI in patients with LEAD.

**Methods:** The subjects were 165 outpatients with LEAD. Arterial blood flow in lower extremities was evaluated by using ABI, TBI, and the degree of leg exercise-induced reduction of ABI (%).

**Results:** CAVI showed significant positive correlations with ABI and TBI and showed significant inverse correlations with exercise-induced % decrease in ABI. CAVI was significantly higher in the 3rd tertile groups of ABI and TBI than that in the corresponding 1st tertile groups and was significantly lower in the 3rd tertile group of exercise-induced % decrease in ABI than that in the 1st tertile group. The above relationships remained significant after adjustment for age, body mass index, blood pressure, diabetes history, and habitual smoking.

**Conclusions:** Although CAVI is a general parameter reflecting arterial stiffness, CAVI showed paradoxical associations, namely, positive associations with ABI and TBI and an inverse association with exercise-induced % decrease in ABI in patients with LEAD.

## Introduction

Lower extremity artery disease (LEAD) is an obstructive disease due to atherosclerosis in arteries of the lower extremities.^1,2)^ Smoking and diabetes are major risk factors of LEAD.^3)^ Measurement of ankle-brachial index (ABI) is a standard examination for diagnosing LEAD in leg arteries, and ABI of 0.9 or lower is generally used as a definition of LEAD.^4)^ ABI of 1.3 or higher is also regarded to be abnormal because of the high pressure needed by sclerosis due to calcification of the arterial wall.^5)^ Toe-brachial index (TBI) is used for diagnosing LEAD in foot arteries. Toe blood pressure is lower than ankle blood pressure and is regarded as abnormal when TBI is less than 0.6–0.7.^6)^

Arterial stiffness is a component of atherosclerosis and is caused by morphological changes in the intimal and medial layers such as decrease in elastic fibers and increase in calcium deposition and by functional changes including increased sympathetic activity and decreased vasorelaxing substances such as nitric oxide and prostacyclin from the endothelium.^7)^ Since atherosclerosis is the pathogenesis of LEAD, arterial stiffness is increased in patients with LEAD.^8)^

Pulse wave velocity (PWV) is often used as an index for evaluating the degree of arterial stiffness. There have been conflicting findings regarding the relationships between ABI and arterial PWV. Aortic PWV (a-PWV), carotid-femoral PWV (ca-PWV), and brachio-ankle PWV (ba-PWV) were shown to be inversely associated with ABI in the general population, patients with coronary artery disease, and hypertensive sibships, respectively.^9–11)^ In a longitudinal study, the rate of change in ca-PWV was inversely associated with the rate of change in ABI.^12)^ Another study showed a U-shaped relationship between a-PWV and ABI in the general population.^13)^ No association was found between ba-PWV and ABI in patients from a cardiology unit^14)^ and in patients with type 2 diabetes.^15)^ On the other hand, a positive longitudinal association was found between ba-PWV and ABI in the general population.^16)^ In patients with LEAD, ABI showed positive associations with ba-PWV and ca-PWV.^17,18)^ Thus, findings in previous studies regarding the relationship between ABI and PWV are controversial, and the relationship is suspected to be affected by the presence of LEAD. Cardio-ankle vascular index (CAVI) is a more recent parameter reflecting stiffness of the arterial tree from the aortic origin to the ankle^19)^ and is calculated by modification of heart–ankle PWV using stiffness parameter β.^20)^ A major merit of CAVI is its independence of blood pressure. A recent study showed that in patients with LEAD, ABI was higher in those with high CAVI than in those without high CAVI.^21)^ Thus, the results of previous studies suggest a positive association between ABI and arterial stiffness evaluated by ba-PWV and CAVI in patients with LEAD, although ABI decreases with progression of atherosclerosis. However, there has been little information on the correlation between ABI and arterial stiffness in patients with LEAD. Moreover, it remains to be clarified whether and how PWV and CAVI are associated with TBI and exercise-induced decrease in ABI.

In this study, correlations of ABI and TBI with CAVI in patients with LEAD were therefore investigated. In addition, we evaluated the decrease in ABI following leg exercise and analyzed its relation with CAVI. ABI, exercise-induced decrease in ABI, TBI, and CAVI were measured in both the right and left legs and feet, and measurements on each side were analyzed separately.

## Materials and Methods

### Study population

Outpatients with LEAD (145 men and 20 women) were the subjects of this study, and their mean age was 74.3 ± 8.0 years. The criteria used for diagnosing LEAD initially were a low ankle-brachial systolic pressure index (ABI ≤0.9) and/or a low toe-brachial systolic pressure index (TBI <0.7).^3,6)^ About 60% of the subjects (n = 98) had already received surgical or endovascular therapy for LEAD.

This study was performed according to the protocol approved by the Ethics Committee of Yamagata Saisei Hospital (approval number: 199 at the ethics committee since 2013). Informed consent was obtained from all the participants. Information of histories of present and previous illness and medication therapy, and habits of cigarette smoking and alcohol consumption was collected by a survey using questionnaires. The subjects were divided into six groups of smokers by using a unit of pack-years, which was calculated by multiplication of the number of packs of cigarettes consumed per day and the number of years each subject had smoked (nonsmokers; 10 pack-years or less; more than 10, and 20 or less pack-years; more than 20, and 40 or less pack-years; more than 40, and 60 or less pack-years; more than 60 pack-years). Smokers were categorized by daily average cigarette consumption as light smokers (20 cigarettes or less) and heavy smokers (21 or more). Habitual alcohol drinking (frequency of weekly drinking) was also surveyed by the questionnaires. Three categories of alcohol drinking used for analysis were regular drinkers (≥5 days per week), occasional drinkers (≤4 days per week), and nondrinkers.

### Measurements

Height (meter) and body weight (kilogram) were measured, and body mass index (BMI) was calculated as weight divided by the square of height.

Venous blood was collected from each subject in the morning after overnight fasting. Concentrations of hemoglobin A_1c_ were determined by using an automatic glyco-hemoglobin analyzer based on high-performance liquid chromatography (ADAMSTM A1c HA-8170; Sekisui Medical Co., Ltd., Tokyo, Japan). Calibration of hemoglobin A_1c_ values was performed using the formula by the Japan Diabetes Society.^22)^ Subjects were diagnosed as diabetic when they were receiving medication therapy for diabetes and/or showed high hemoglobin A_1c_ levels of 6.5% or higher, which are the criteria proposed by the American Diabetes Association.^23)^

After a quiet rest in a supine position, ABI and TBI levels at rest were determined by an oscillometric method using automatic ABI and TBI devices (VaSera VS-1500; Fukuda Denshi, Tokyo, Japan). ABI was also measured after loading leg stress with fatigue in the gastrocnemius and soleus muscles, which was induced by isotonic ankle-plantar-flexion exercise (100 pedals for each leg at 60 beats per minute in an alternate basis that correspond to 5.3 joules of work) using a leg loader, a stress-loading device (VSL-100A; Fukuda Denshi).^24)^ Leg exercise was performed in all the subjects. CAVI and blood pressure of the brachial arteries were also recorded by using the above instrument (VaSera VS-1500). In multivariable analyses, mean arterial pressure level, defined as the diastolic blood pressure level plus one-third of the difference between systolic and diastolic blood pressure levels, was used as a variable for adjustment.

### Statistical analysis

A computer software program (IBM SPSS Statistics for Windows, Version 25.0.; IBM Corp, Armonk, NY, USA) was used for statistical analyses. Categorical variables were displayed as frequencies and percentages. Continuous variables that showed normal distributions were displayed as means with standard deviations or means with 95% confidence intervals. In univariable correlation analyses, Pearson’s correlation coefficients and Spearman’s rank correlation coefficients were calculated in analyses of variables showing normal distributions and variables not showing normal distributions, respectively. Standardized partial regression coefficients were calculated in multivariable analysis of linear regression when variables showed normal distributions. Tertile groups of each of three variables (ABI, exercise-induced decrease in ABI and TBI) were prepared as follows: values of all subjects were arranged in an ascending order, and then, they were divided into three tertile groups of approximately equal sizes. Mean levels of each variable were compared among tertile groups by using analysis of variance followed by the Scheffé’s F-test as a post-hoc test in univariable analysis and analyses of covariance followed by the Student’s t-test after Bonferroni correction in multivariable analyses. In multivariable analyses, adjustment was performed using the following explanatory variables and covariates: age, sex, adiposity (BMI), blood pressure (mean arterial pressure), a diabetes history, lifestyles such as cigarette smoking and alcohol drinking, and histories of medication therapy using insulin and anticoagulants. Statistical significance was considered when probability (*p*) values were less than 0.05.

## Results

### Characteristics of the subjects

**Table 1** shows profiles of the subjects. Eighty-eight percent of the subjects were men, and the mean age of the subjects was 74.3 years. The proportions of smokers and patients with diabetes were 24.2% and 41.2%, respectively. The mean values of CAVI were 9.23 (right) and 9.05 (left), the mean values of ABI were 0.876 (right) and 0.894 (left), and the median values of TBI were 0.64 (right) and 0.65 (left).

**Table 1:** Characteristics of subjects with LEAD

Variables	Values
Gender	145 men and 20 women
Age	74.3 ± 8.0
Rutherford classification	Category 1, n = 78; category 2, n = 87
Smokers (%)	24.2 (light, 21.2; heavy, 3.0)
Alcohol drinkers (%)	56.4 (occasional, 19.4; regular, 37.0)
History of diabetes (%)	41.2
History of insulin therapy (%)	6.7
History of anticoagulation therapy (%)	79.4
BMI (kg/m^2^)	22.9 ± 3.0
Systolic blood pressure (mmHg)	134.6 ± 14.3
Diastolic blood pressure (mmHg)	71.1 ± 11.0
Mean arterial pressure (mmHg)	92.3 ± 10.6
Hemoglobin A_1c_ (%)	6.30 ± 0.97
CAVI (right)	9.23 ± 2.29
CAVI (left)	9.05 ± 2.06
ABI (right)	0.876 ± 0.189
ABI (left)	0.894 ± 0.192
TBI (right)	0.64 (0.45, 0.81)
TBI (left)	0.65 (0.51, 0.79)
% decrease in ABI by exercise (right)	16.7 ± 14.2
% decrease in ABI by exercise (left)	16.3 ± 16.1

Shown are numbers (n), proportions, means with standard deviations, and medians with 25 and 75 percentile values in parentheses.

LEAD: lower extremity artery disease; BMI: body mass index; CAVI: cardio-ankle vascular index; ABI: ankle-brachial index; TBI: toe-brachial index

### Correlations between CAVI and indices of leg and foot arterial flow

Correlation coefficients of CAVI with ABI, TBI, and % decrease in ABI induced by leg exercise are shown in **Table 2**. Univariable and multivariable analyses were performed for calculating the correlation coefficients of CAVI with ABI and % decrease in ABI after leg exercise. In multivariable analysis, age, gender, history of diabetes, habit of smoking, and mean arterial pressure were used as explanatory variables (Multivariable-1 in the table). In addition, BMI, habit of alcohol drinking, and histories of insulin therapy and anti-coagulation therapy were included in explanatory variables in the other multivariable analysis (Multivariable-2 in the table). Since TBI could not be measured in some patients due to severe stenotic arterial lesions, only Spearman’s rank correlation coefficients were calculated for the relationships between CAVI and TBI.

**Table 2:** Correlations between CAVI and each variable of leg arterial flow in univariable analysis and multivariable analysis

	Univariable	Multivariable-1	Multivariable-2
ABI (right)	0.364**	0.410**	0.422**
ABI (left)	0.328**	0.380**	0.388**
% decrease in ABI by exercise (right)	−0.331**	−0.394**	−0.390**
% decrease in ABI by exercise (left)	−0.340**	−0.351**	−0.358**
TBI (right)	0.172*	n.d.	n.d.
TBI (left)	0.256**	n.d.	n.d.

For univariable analysis, Pearson’s correlation coefficients of CAVI with ABI and % decrease in ABI induced by exercise and Spearman’s rank correlation coefficients of CAVI with TBI are shown. For multivariable analysis, standardized partial regression coefficients between each of the above pairs are shown. Age, gender, habit of smoking, history of diabetes, and mean arterial pressure were adjusted in the multivariable analysis (Multivariable-1). In addition, habit of alcohol drinking, histories of insulin therapy and anticoagulation therapy, and BMI were used as other explanatory variables in the other multivariable analysis (Multivariable-2). Asterisks denote significant correlation coefficients (*, *p* <0.05; **, *p* <0.01).

CAVI: cardio-ankle vascular index; ABI: ankle-brachial index; TBI: toe-brachial index; n.d., not determined

**Figure 1:**
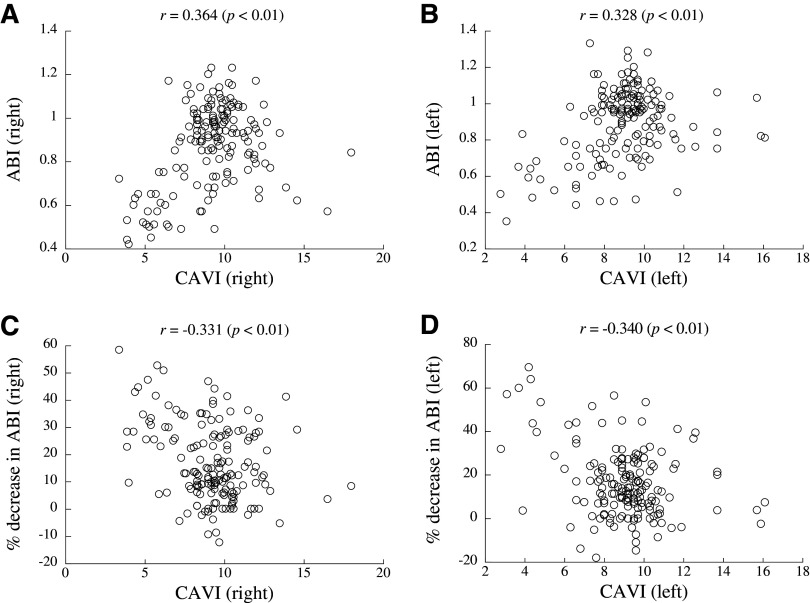
Fig. 1 Scatter plots of the relationships of CAVI with ABI (**A** and **B**) and % decrease in ABI induced by leg exercise (**C** and **D**). Pearson’s correlation coefficients (r) with *p* values are shown in the figures. CAVI: cardio-ankle vascular index; ABI: ankle-brachial index.

In univariable analyses, CAVI showed significant positive correlations with ABI and TBI and showed significant inverse correlations with % decrease in ABI induced by leg exercise. Scatter plots of the relationships of CAVI with ABI and % decrease in ABI induced by leg exercise are shown in **Fig. 1**. The correlation coefficients of CAVI with ABI and % decrease in ABI induced by leg exercise tended to be higher than those of CAVI with TBI. In multivariable analyses with adjustments for the above variables, CAVI was also significantly correlated with ABI and % decrease in ABI induced by leg exercise. The standardized partial regression coefficients with adjustment for the above nine variables were 0.422 (right) and 0.388 (left) between CAVI and ABI and they were −0.390 (right) and −0.358 (left) between CAVI and % decrease in ABI induced by exercise.

### Comparisons of CAVI in tertile groups of each index of leg and foot arterial flow

CAVI was compared among the three tertile groups of each of the indices including ABI, TBI, and % decrease in ABI induced by exercise (**Table 3**). CAVI was significantly higher in the 3rd tertile groups of ABI and TBI than that in the corresponding 1st tertile groups and was significantly lower in the 3rd tertile group of exercise-induced % decrease in ABI than that in the corresponding 1st tertile group. CAVI was also significantly higher in the 2nd tertile group of ABI than that in its 1st tertile. The above results were obtained both in right-side and left-side measurements and both in univariable and multivariable analyses (Multivariable-1 and Multivariable-2) as shown in **Table 3**.

**Table 3:** Comparisons of CAVI among the tertile groups of ABI, % decrease in ABI induced by exercise, and TBI in univariable and multivariable analyses

	Univariable	Multivariable-1	Multivariable-2
ABI (right) 1st (n = 56) 2nd (n = 56) 3rd (n = 53)	8.29 (7.46–9.13) 9.84 (9.35–10.33)** 9.58 (9.26–9.90)*	8.12 (7.55–8.68) 9.86 (9.30–10.42)** 9.74 (9.16–10.32)**	8.07 (7.52–8.62) 9.41 (8.90–9.93)** 9.68 (9.12–10.24)**
ABI (left) 1st (n = 53) 2nd (n = 60) 3rd (n = 52)	8.17 (7.44–8.91) 9.46 (9.02–9.90)** 9.48 (9.10–9.86)**	8.07 (7.53–8.61) 9.38 (8.88–9.88)** 9.68 (9.13–10.23)**	7.89 (7.31–8.46) 9.40 (8.86–9.93)** 9.67 (9.08–10.26)**
% decrease in ABI by exercise (right) 1st (n = 55) 2nd (n = 55) 3rd (n = 55)	9.90 (9.34–10.45) 9.39 (8.90–9.87) 8.41 (7.67–9.14)**	10.02 (9.44–10.60) 9.34 (8.76–9.92) 8.33 (7.76–8.90)**	10.03 (9.43–10.62) 9.35 (8.76–9.94) 8.34 (7.76–8.93)**
% decrease in ABI by exercise (left) 1st (n = 55) 2nd (n = 55) 3rd (n = 55)	9.63 (9.04–10.21) 9.17 (8.66–9.67) 8.41 (7.74–9.08)**	9.65 (9.11–10.19) 9.11 (8.58–9.65) 8.39 (7.85–8.94)**	9.68 (9.13–10.23) 9.10 (8.55–9.65) 8.42 (7.87–8.97)**
TBI (right) 1st (n = 53) 2nd (n = 57) 3rd (n = 55)	8.59 (7.76–9.42) 9.13 (8.82–9.43) 9.70 (9.33–10.07)*	8.43 (7.83–9.04) 9.47 (8.89–10.04)* 9.75 (9.16–10.34)**	8.46 (7.85–9.07) 9.46 (8.87–10.05) 9.76 (9.16–10.36)*
TBI (left) 1st (n = 55) 2nd (n = 55) 3rd (n = 55)	8.35 (7.68–9.02) 9.17 (8.66–9.67) 9.64 (9.23–10.06)**	8.23 (7.69–8.76) 9.26 (8.73–9.79)* 9.67 (9.14–10.21)**	8.19 (7.65–8.73) 9.32 (8.78–9.87)* 9.68 (9.14– 10.21)**

Shown are means of CAVI with its 95% confidence intervals. Age, gender, habit of smoking, history of diabetes, and mean arterial pressure were adjusted in the multivariable analysis (Multivariable-1). In addition, habit of alcohol drinking, histories of insulin therapy and anticoagulation therapy, and BMI were used as explanatory variables in the other multivariable analysis (Multivariable-2). Asterisks denote significant differences from the 1st tertile group of each variable (*, *p* <0.05; **, *p* <0.01).

CAVI: cardio-ankle vascular index; ABI: ankle-brachial index; TBI: toe-brachial index; BMI: body mass index

### Correlations of absolute difference in right and left CAVI levels with ABI and exercise-induced decrease in ABI

The median value of the absolute difference in right and left CAVI levels was 0.8 (25 and 75 percentile values: 0.3 and 2.0). There was a significant inverse correlation between the absolute difference in CAVI levels and mean ABI of right- and left-side measurements (Spearman’s rank correlation coefficient: −0.472 [*p* <0.01]). The absolute difference in CAVI levels showed a significant positive correlation with the mean exercise-induced % decrease in ABI (Spearman’s rank correlation coefficient: 0.175 [*p* <0.05]). Since either right or left TBI or both right TBI and left TBI could not be measured in 16 subjects, data for the remaining 149 subjects were used for calculation of the correlation coefficient between the absolute difference in CAVI levels and mean TBI, which was −0.230 (*p* <0.01).

### Comparison of CAVI and ABI in the two groups of subjects with category 1 and category 2 of the Rutherford classification

The subjects of this study showed category 1 or category 2 of the Rutherford classification (**Table 1**), and individuals with other categories (categories 3–6) of the classification were not included in the subjects. We compared CAVI and ABI levels of the two subject groups with category 1 and category 2 of the Rutherford classification. As shown in **Table 4**, both CAVI and ABI were significantly lower in the subject group with category 2 of the Rutherford classification than in the category 1 group.

**Table 4:** Comparisons of CAVI and ABI in subject groups with category 1 and category 2 of the Rutherford classification

	Rutherford classification		
	Category 1 (n = 78)	Category 2 (n = 87)
CAVI Univariable Multivariable-1 Multivariable-2	9.65 (9.35–9.96) 9.66 (9.27–10.06) 9.70 (9.30–10.11)	8.50 (8.05–8.95)** 8.49 (8.12–8.87)** 8.48 (8.10–8.86)**
ABI Univariable Multivariable-1 Multivariable-2	0.940 (0.910–0.969) 0.938 (0.904–0.972) 0.941 (0.906–0.977)	0.836 (0.799–0.872)** 0.837 (0.805–0.869)** 0.834 (0.801–0.867)**

Means with 95% confidence intervals are shown. Mean levels of CAVI and ABI were compared in the two groups of subjects with categories 1 and 2 of the Rutherford classification. Age, gender, habit of smoking, history of diabetes, and mean arterial pressure were adjusted in the multivariable analysis (Multivariable-1). In addition, habit of alcohol drinking, histories of insulin therapy and anticoagulation therapy, and BMI were used as explanatory variables in the other multivariable analysis (Multivariable-2). Asterisks denote significant differences (**, *p* <0.01) from the group of subjects with category 1 of the Rutherford classification.

CAVI: cardio-ankle vascular index; ABI: ankle-brachial index; BMI: body mass index

## Discussion

In this study, CAVI was demonstrated to be positively and inversely associated with ABI and exercise-induced decrease in ABI, respectively. Those associations were not changed after adjustment for age, gender, BMI, blood pressure, habits of smoking and alcohol drinking, history of diabetes, and histories of medication therapy using insulin and anticoagulants. The above findings regarding the relationships between ABI and CAVI agree with the results of a recent study showing that ABI was significantly higher in LEAD patients with high CAVI (>9) than in those without high CAVI.^21)^ In addition, the present study showed for the first time that CAVI was inversely associated with exercise-induced decrease in ABI and was positively associated with TBI. Decrease in ABI induced by leg exercise is a clinically useful variable for detecting reduction of arterial blood flow in lower extremities at an early stage of patients with LEAD and evaluating severity of the disease. With an advance of atherosclerotic lesion, arterial blood flow is decreased due to stenotic lesions, resulting in a decrease in blood pressure of leg arteries when leg exercise is loaded. Thus, % decrease in ABI induced by leg exercise is inversely associated with ABI. Accordingly, it is reasonable that % decrease in ABI induced by leg exercise as well as ABI showed a paradox in its association with CAVI in patients with LEAD.

Both ABI and TBI showed positive associations with CAVI. Decreases in ABI and TBI are known as signs of reduced blood flow in leg and foot arteries, which is accelerated by progression of atherosclerosis. Arterial stiffness, i.e., arteriosclerosis, is a manifestation of atherosclerosis.^7)^ Therefore, CAVI does not reflect arterial stiffness in patients with LEAD. CAVI was lower in subjects with higher severity of leg ischemia (category 2 of the Rutherford classification) than that in those with lower severity of ischemia (category 1). This result agrees with the other paradoxical findings in the present study that CAVI was positively associated with ABI and TBI. On the other hand, ABI was lower in subjects with higher severity of ischemia than that in subjects with lower severity of ischemia, indicating that a decrease in ABI reflects severity of ischemia in patients with LEAD. Thus, the degree of symptomatic leg ischemia evaluated by the Rutherford classification coincided with the results of ABI but was opposite to the results of CAVI.

In patients with severe stenosis or occlusion of affected leg and foot arteries, collateral circulation is formed, causing a slower arterial flow velocity, i.e., retarded propagation of the pulse wave. This may be the reason for lower CAVI in patients with more severe lesions, which accompany more collateral circulation, resulting in positive associations of CAVI with ABI and TBI and inverse associations of CAVI with degree of leg ischemia and % decrease in ABI induced by leg exercise. Another possible reason for the positive associations of CAVI with ABI and TBI was their increases in affected arteries due to calcification of the vascular wall by advanced atherosclerosis. However, this reason is unlikely because there were significant positive correlations (*p* <0.01) between CAVI and ABI when analyzing only the subjects showing low ABI (≤0.9) (Pearson’s correlation coefficients: right, 0.457; left, 0.502). We also investigated the correlations between ABI and CAVI in subjects showing relatively high ABI (>0.9): Pearson’s correlation coefficients between ABI and CAVI were −0.101 (*p* = 0.350) (right) and −0.038 (*p* = 0.718) (left). Thus, there was no association between ABI and CAVI in the subject group showing relatively high ABI, in which the lesions with relatively high degree of calcification are suspected to exist. Therefore, in those patients, both collateral circulation resulting from arterial stenosis and ectopic calcification, which retardates and accelerates pulse wave propagation, respectively, affect the CAVI levels, resulting in no association between ABI and CAVI. Moreover, there is also a possibility that atherosclerotic lesions with a high degree of calcification disturb accurate measurement of arterial pressure at the ankle joint level. Interestingly, ca-PWV has been shown to be lower in LEAD patients who had aorto-iliac disease than in those without aorto-iliac disease.^18)^ This suggests that PWV becomes slower when the site of the arterial lesion is overlapped with the pathway of pulse wave transmission. In the present study, the association between ABI and CAVI tended to be stronger than that between TBI and CAVI. This finding may be reasonable since CAVI reflects the velocity of arterial flow between brachial and ankle arteries, ABI is a ratio of ankle blood pressure to brachial blood pressure, and the toe artery is more peripheral than the ankle artery.

In a previous study by Yokoyama et al.,^17)^ the absolute difference in right and left baPWV levels showed a significant inverse correlation with ABI in nine diabetes patients with LEAD (Spearman’s rank correlation coefficient: −0.783). Similarly, the absolute difference in right and left CAVI levels showed a significant inverse correlation with mean ABI (Spearman’s rank correlation coefficient: −0.472 [*p* <0.01]) in the present study. Moreover, the absolute difference in CAVI levels showed a significant inverse correlation with TBI and a significant positive correlation with exercise-induced decrease in ABI. Thus, the absolute difference in right and left CAVI levels reflects the severity of stenosis in affected arteries in patients with LEAD. Moreover, the absolute difference in right and left baPWV levels has been shown to be significantly higher in diabetes patients with LEAD than in those without LEAD.^15,17)^ Therefore, absolute differences in right and left levels of baPWV and CAVI may be useful parameters for diagnosing LEAD. Future studies using data of the general population and LEAD patients before any medical therapy are needed to clarify the clinical significance of the difference between right and left CAVI levels as a parameter for diagnosing LEAD.

## Study limitations

The mean age of the subjects was 74.3 years and thus most of them were elderly patients with LEAD. Further studies using databases of younger patients are needed to confirm the findings of this study. The subjects were outpatients who had already received therapy for LEAD. Thus, analysis of a database of patients before any kind of therapy is needed to determine the precise pathophysiology of the relation of ABI with PWV. Since this study is cross-sectional in its design, clinical trials including intervention therapy for LEAD are needed to discuss causality of the relationship between ABI and CAVI. Interestingly, both ba-PWV and ABI have been reported to be increased after successful arterial dilatation by percutaneous transluminal angioplasty in diabetes patients with LEAD.^17)^ This suggests that ba-PWV was reduced by stenosis of leg arteries in LEAD patients and that collateral circulation might be decreased by angioplasty therapy.

## Conclusion

Although arterial stiffness is increased in patients with LEAD, CAVI showed positive associations with ABI and TBI and showed inverse associations with degree of leg ischemia and exercise-induced % decrease in ABI. Thus, there is a paradox regarding the relationships of CAVI with ABI and TBI. This means that CAVI as well as PWV cannot be used for evaluation of arterial stiffness in patients with LEAD. Instead, the absolute difference of CAVI values of right- and left-side measurements is proposed as an index for evaluating the severity of LEAD. Vascular wall lesions in leg and foot arteries are thought to result in retardation of pulse wave propagation in patients with LEAD. This may be caused by formation of collateral circulation, in which blood flow is slower than that in affected arteries.

## IRB Information

This study was performed according to the protocol approved by the Ethics Committee of Yamagata Saisei Hospital (approval number: 199 at the ethics committee since 2013). Informed consent was obtained from all the subjects.

## Funding

This study was supported by a Grant-in-Aid for Scientific Research (No. 21H03386) from the Japan Society for the Promotion of Science (to IW).

## Disclosure Statement

All the authors have stated they have no relevant relationships to disclose about the contents of this paper.

## Author Contributions

Study conception: YS and IW

Data collection: YS, SH, and HO.

Analysis: YS and IW

Investigations: all authors

Writing: IW

Critical review and revision: all authors

Final approval of the article: all authors

Accountability for all aspects of the work: all authors.
